# Modification and Enhancing Contribution of Fiber to Asphalt Binders and Their Corresponding Mixtures: A Study of Viscoelastic Properties

**DOI:** 10.3390/ma16165727

**Published:** 2023-08-21

**Authors:** Chao Li, Hao Liu, Yue Xiao, Jixin Li, Tianlei Wang, Longfan Peng

**Affiliations:** 1 School of Materials Science and Engineering, Wuhan University of Technology, Wuhan 430070, China; lchao@whut.edu.cn (C.L.); wangtianlei@whut.edu.cn (T.W.); 2 China Construction Second Engineering Bureau Ltd., Beijing 100176, China; liuhao-2@cscec.com (H.L.); lijixin@cscec.com (J.L.); penglongfan@cscec.com (L.P.); 3 School of Materials Science and Engineering, Chang’an University, Xi’an 710018, China

**Keywords:** shear resistance, fiber-reinforced asphalt mixture, viscoelastic property, resilience modulus, master curves

## Abstract

The performance of asphalt binders and asphalt mixtures can be enhanced by the inclusion of fiber. The viscoelastic characteristics of fiber-reinforced asphalt binders and their corresponding mixtures were characterized in this study. To generate fiber-reinforced asphalt samples for dynamic shear rheometer (DSR) tests, polypropylene fibers (PPFs), polyester fibers (PFs), and lignin fibers (LFs) were added into modified asphalt with a ratio of 5wt%. Indirect tensile resilience tests were conducted on the fiber-reinforced asphalt mixture with Marshall samples, which was prepared with a 6.4% of bitumen/aggregate ratio. The addition of fiber can increase the anti-rutting performance of asphalt binders, and also reduce the anti-fatigue performance of asphalt binders to varying degrees. Viscoelastic properties of the fiber-reinforced asphalt binders are highly dependent on the shape of the used fiber. The resistance of the fiber-reinforced asphalt binders to rutting at high temperatures increases with the roughness degree of the fiber’s surface morphology. PPF-reinforced asphalt binders surpass the others in terms of anti-rutting capabilities. The high-temperature deformation resistance of the PPF-reinforced asphalt mixture is stronger, whereas the low-temperature crack resistance of the PF-reinforced asphalt mixture is stronger, which can be observed from the master curve of indirect tensile resilient modulus.

## 1. Introduction

With the development of the automobile industry, the demand for high-speed vehicle test sites has been increasing. The high-speed vehicle test field is a place for high-speed tests of vehicle roads, including various test roads such as annular runways and ramps for high-speed driving of vehicles [1]. The vehicle loading conditions on the high-speed vehicle test road are not heavy, but the friction force applied by the car during high-speed driving is very large, which makes the high-speed vehicle test road bear a large shear force. The shear force of the pavement is too large, which will lead to pavement cracking, aggregate loss, rutting, and other damage, which will seriously affect the durability of asphalt pavement [2]. It is found that fiber, as a high-strength material, can significantly improve the viscoelastic properties of asphalt mixture and prolong the service life of asphalt pavement [3,4,5,6,7].

Nowadays, lignin fiber, polyester fiber, polypropylene fiber, glass fiber, etc., are the most often used fibers in road engineering. Several qualities of the asphalt mixture are enhanced to varying degrees by different fibers [8,9,10,11,12]. In recent years, the enhancement of fiber on the viscoelastic properties of asphalt binders has been the main focus of research on fiber-reinforced asphalt mixtures [13,14]. Wang used ceramic fiber to prepare a fiber-reinforced asphalt mixture. The wheel tracking test, Marshall immersion test, and freeze–thaw splitting test showed that ceramic fiber could improve the viscoelastic properties of the asphalt mixture; the optimum content of fiber was 0.4% [15]. Chen used fiber from corn straw as a reinforcing material. It was discovered through rotating viscosity testing and bending beam rheometer testing that the inclusion of corn straw fiber could improve the viscosity of asphalt binder and decrease its sensitivity to temperature [16]. Eltwati added glass fiber to an RAP asphalt mixture. Through the Hamburg wheel track test, it was found that glass fiber enhances the rutting resistance of an RAP asphalt mixture; the optimum content of glass fiber was 0.2% [17]. Xia studied the durability of a bamboo fiber asphalt mixture from three aspects: aging durability, freeze–thaw cycle durability, and fatigue durability. The results show that adding bamboo fiber can effectively improve the durability of an asphalt mixture [18].

Most of the research on fiber as a reinforcing agent focuses on macroscopic mechanical properties, and few studies explain the internal causes of fiber-reinforced asphalt mixture performance from a microscopic perspective [19,20,21,22,23]. Liu analyzed the adsorption of fibers on asphalt binders and the enhancement of the mixture by scanning electron microscopy (SEM). It was observed that the fiber and asphalt had good wettability and dispersibility in the mixture [24]. Miao made use of polymer fiber, lignin fiber, basalt fiber, and various types of asphalt. The impact of asphalt and fiber surface characteristics on the functionality of fiber-modified asphalt binders was examined by evaluating the surface energy parameters of the fiber and asphalt binders and the shear strength of the fiber-modified asphalt binders [25]. Hong discovered that the performance of the reinforced asphalt mixture may be coordinated by the filler and fiber-reinforcing agent in the asphalt mixture [26].

Nevertheless, most of the research on fiber-reinforced asphalt mixtures only focuses on one fiber and only focuses on the performance of fiber-reinforced asphalt mixtures. The use of multiple fibers to compare the effects of fiber types on fiber-reinforced asphalt mixtures was not considered. Moreover, the combination of fiber-reinforced asphalt binders and fiber-reinforced asphalt mixtures is not considered to illustrate the role of fiber. Therefore, the application of fibers in asphalt mixtures has not been widely used due to the lack of a viscoelastic enhancement mechanism study.

In this study, PFs and PPFs were selected as the representative of polymer fiber, and LFs were selected as the representative of organic fiber to study the relationship between the fiber type of SMA-13 fiber-reinforced asphalt mixture and the viscoelastic properties of asphalt binders and asphalt mixture. Korean SK Speedway-modified asphalt was used as a binder to prepare fiber-reinforced binders. The relationship between rutting parameters, fatigue parameters, and fiber types of fiber-reinforced asphalt binders was analyzed by the DSR test with temperature scanning mode. Through the indirect tensile test of the Marshall specimen of asphalt mixture, the indirect tensile resilient modulus is measured, and the master curve of resilient modulus is drawn by the principle of time–temperature equivalence, so as to analyze the influence of fiber type on the viscoelastic properties of an asphalt mixture. In addition, the fiber was tested by SEM, and the internal factors of the influence of fiber type on the viscoelastic properties of asphalt binders and asphalt mixture were analyzed from the perspective of microstructure. The purpose of this paper is to analyze the influence of fiber types on the viscoelastic properties of asphalt binders and asphalt mixtures. On this basis, the viscoelastic properties of fiber-reinforced asphalt binders and fiber-reinforced asphalt mixtures are combined and analyzed, which can provide a factual basis for the selection of fiber-reinforced agents for anti-cracking and anti-rutting pavement. It has a practical guiding role in the construction of high-speed vehicle test pavement.

## 2. Materials and Methodologies

### 2.1. Materials

#### 2.1.1. Fibers

**Figure 1:**
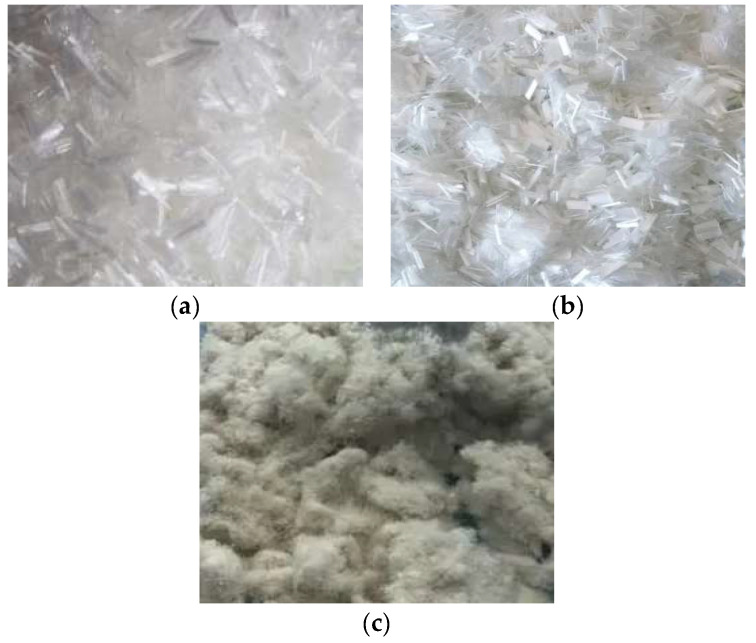
Used fibers: (**a**) PPFs, (**b**) PFs, (**c**) LFs.

PFs, LFs, and PPFs produced by Hubei Lu Xiang Chemical Industry were selected for the fiber-reinforced asphalt mixture design and performance analysis. The intuitive morphology of the three involved fibers is shown in Figure 1. It can be seen from the figure that PPFs and PFs are glossy white bundle fibers, and LFs are gray flocculent fibers.

**Figure 2:**
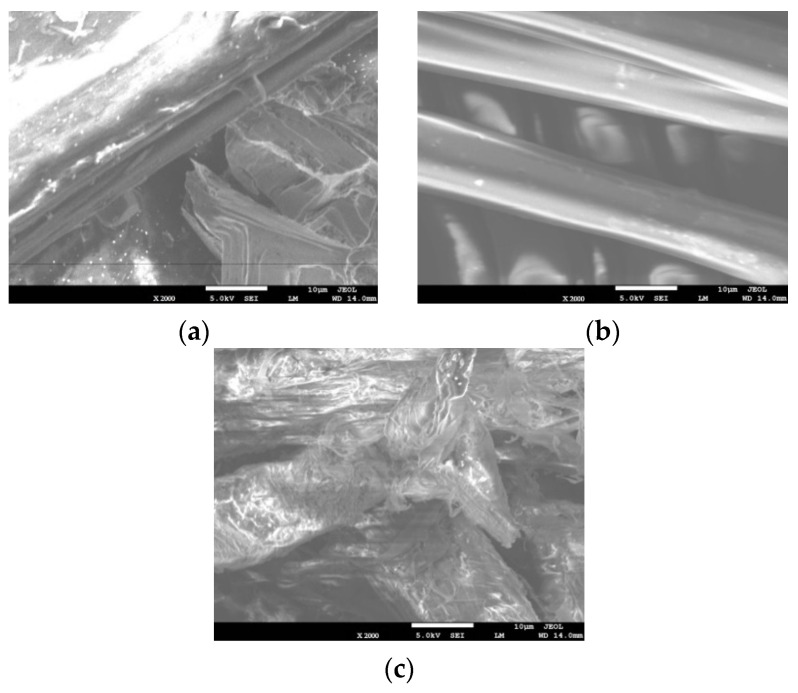
SEM images of fibers: (**a**) PPFs, (**b**) PFs, (**c**) LFs.

In order to understand the surface structure of the used fibers, SEM was conducted, and the test results are shown in Figure 2. These images were taken at a voltage of 15 kV and a magnification of 2000 times. These images can fully show the micro-morphology of the fibers. It can be seen that the surface of PFs is the smoothest and the fiber morphology is more regular. PPFs followed, and although their surface is not smooth, their structure is also regular. The surface of LFs is the roughest and the shape is irregular. It can be seen from some cross-section images that the interior of LFs is a layered hollow structure.

According to the requirements of China asphalt pavement fiber standard (JTT 533-2020), the tensile strength of the fiber is tested. The test results are shown in Table 1. For LFs, the oil absorption rate index is of interest. The oil absorption rate of LFs used in this experiment is 6.2 times, which meets the test standard. The performance indexes of PFs and PPFs meet the requirements of test standards, and the performance indexes of PFs are greater than those of PPFs.

#### 2.1.2. Asphalt Binders

The asphalt raw material used in this research is Korean SK-modified asphalt. Penetration test, softening point test, and ductility test: these tests were conducted according to the Standard Test Methods of Bitumen and Bituminous Mixtures for Highway Engineering (JTG E20-2011), with the test results as shown in Table 2. The test results were evaluated by the Technical Specifications for Construction of Highway Asphalt Pavements (JTG E40-2004).

In order to study the influence of different fibers on the viscoelastic properties of asphalt binders, fiber-reinforced asphalt binders were prepared in this experiment. In this test, the content of fiber in fiber-reinforced asphalt binder is 5wt%. Firstly, the fiber was dried to constant weight at 105 °C, and was then added to the flowing SK-modified asphalt at 165 °C to ensure it was evenly mixed.

#### 2.1.3. Asphalt Mixture

In order to study the influence that fibers have on the viscoelastic properties of asphalt mixture, fiber-reinforced asphalt mixture was prepared for this experiment. In this experiment, the discontinuous graded asphalt mastic gravel mixture (SMA) was used, and the proportion of coarse aggregate in SMA was as high as 70–80% [27,28]. Due to the good interlocking of coarse aggregate, SMA usually uses fiber as a stabilizer. Therefore, SMA usually has good high-temperature rutting resistance and low-temperature deformation resistance. SMA is usually used as the surface layer of the extreme-speed competition section. In this study, the SMA-13 asphalt mixture was selected. The blending ratio of various mineral materials in asphalt mixture is (10–15) mm: (5–10) mm: (0–5) mm: mineral powder = 35%:35%:19%:11%. The passing rate of sieve sizes at all levels of gradation A is shown in Table 3.

The mineral aggregate was weighed according to the gradation A, and the content of asphalt binder in asphalt mixture is 6.0%. The Marshall specimens were formed by double-sided compaction 50 times. According to the Standard Test Methods of Bitumen and Bituminous Mixtures for Highway Engineering (JTG E20-2011), the volume properties of the asphalt mixture measured are shown in Table 4. Adding 0.3% fiber to asphalt mixture.

### 2.2. Methodologies

**Figure 3:**
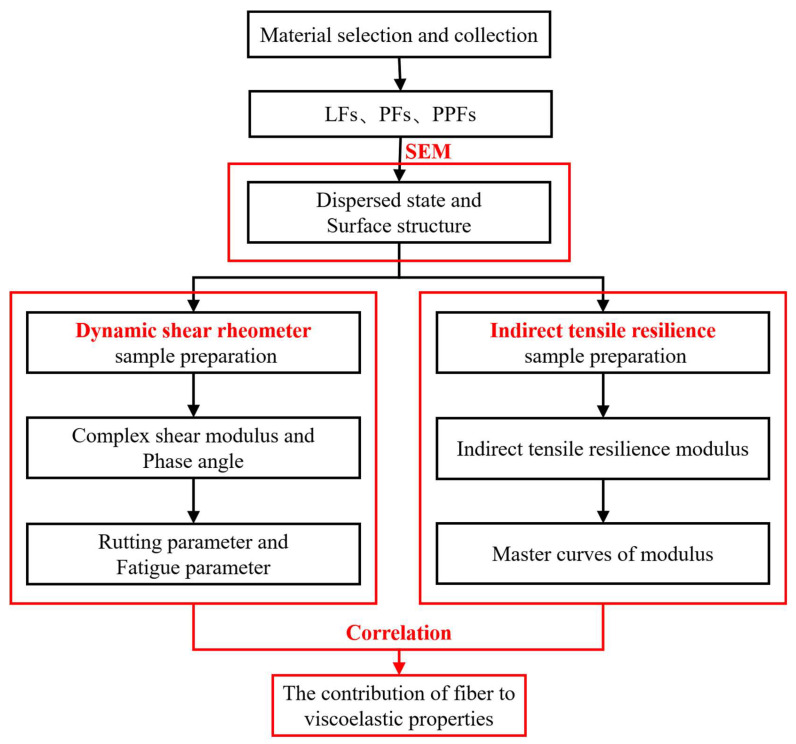
Technical route of this research.

The technical roadmap of this study is explained in Figure 3. Firstly, three kinds of fibers, PFs, PPFs, and LFs, were selected and uniformly mixed with asphalt binders, and the DSR test was carried out. Then, the effects of different fibers on the viscoelastic properties of asphalt binders were analyzed. The Marshall specimens of SMA-13 asphalt mixture were prepared by three kinds of fibers and limestone aggregate, and the indirect tensile resilient modulus was tested. The influence of fiber on the viscoelastic properties of asphalt mixture was analyzed by drawing the time–temperature equivalent master curve. The correlation between fiber and the viscoelastic properties of asphalt binders and asphalt mixtures can therefore be found.

#### 2.2.1. Dynamic Shear Rheometer

The rheological properties of asphalt mastic can be tested by dynamic shear rheometer (DSR). The principle of DSR is to apply sinusoidal oscillating stress or strain to asphalt binders at a specific temperature and loading frequency. The standard DSR test procedure needs to first heat the asphalt binders to the viscous flow state, then pour the asphalt binders into the center of test plate, and adjust the gap between test plate to the standard gap. In the DSR test, there are main two test procedures: frequency scanning and temperature scanning. The tests must be carried out in the linear viscoelastic range of the asphalt binders.

According to Standard Test Methods of Bitumen and Bituminous Mixtures for Highway Engineering (JTG E20-2011), the complex shear modulus and phase angle of fiber-reinforced asphalt binders were obtained by DSR test. Through calculation, the rutting parameter and fatigue parameter of fiber-reinforced asphalt binders were obtained to evaluate the high-temperature deformation resistance and low-temperature cracking resistance of fiber-reinforced asphalt binders. In this experiment, a rotary rheometer produced by Anton Par Co., Ltd. of Austria was used. The instrument model was MCR-302. The instrument can measure the viscosity–temperature curve of various materials in the temperature range from −40 °C to 200 °C. The test conditions of this test are shown in Table 5.

#### 2.2.2. Indirect Tensile Resilience

The dynamic test of asphalt mixture from the perspective of dynamic mechanics can provide a more sufficient reference for the comprehensive investigation of the crack resistance of asphalt mixture. The resilient modulus of asphalt mixture indicates the response of asphalt mixture to repeated load, and it is also the design parameter of pavement structure. It can be used to evaluate the deformation ability and crack resistance of asphalt mixture pavement under normal use [29].

According to the Standard Test Methods of Bitumen and Bituminous Mixtures for Highway Engineering (JTG E20-2011), the viscoelastic properties of fiber-reinforced asphalt mixture were analyzed by indirect tensile resilience test. This test uses the dynamic asphalt mixture test system produced by CONTROLS company. The instrument model is UTM-130, which can provide a force between 15 kN and 130 kN, and can complete most of the stiffness modulus, dynamic modulus, fatigue cracking, hot cracking, and permanent deformation tests.

The indirect tensile modulus of resilience tests was conducted at −10 °C, 0 °C, 20 °C, 35 °C, and 50 °C, with loading frequencies of 10 Hz, 5 Hz, 1 Hz, 0.5 Hz, and 0.2 Hz. The loading time accounts for 1/5 of the total period, and the intermittent time accounts for 4/5 of the total period.

## 3. Viscoelastic Properties of Fiber-Reinforced Binders

The viscoelastic properties of asphalt binders at high and low temperatures were tested by DSR. By measuring the complex shear modulus (G*) and phase angle (δ) of the binders at a set temperature, the obtained results device can predict the behavior images of asphalt pavement at various temperatures during its filed applications. The main purpose of this test is to study the temperature dependence of viscoelasticity of fiber-reinforced asphalt binders, to study the influence of fiber on the viscoelasticity of asphalt binders, and to evaluate the anti-rutting resistance and fatigue resistance of different fiber-reinforced asphalt binders.

### 3.1. Temperature Dependency

#### 3.1.1. Complex Shear Modulus

**Figure 4:**
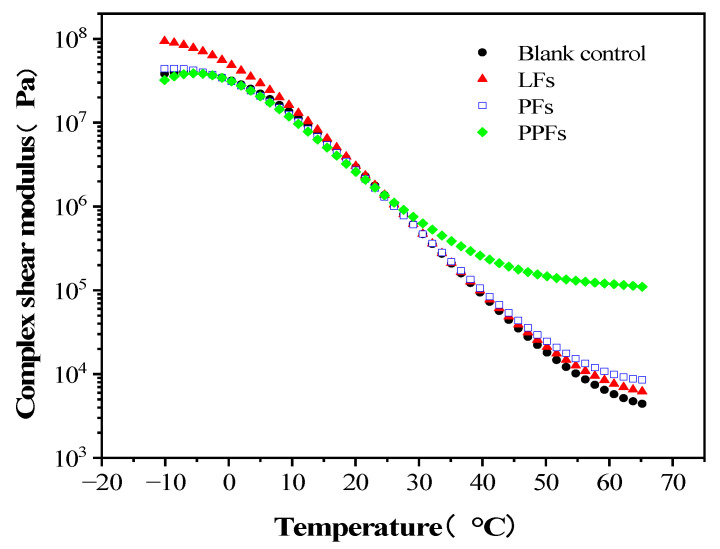
Changing trend of complex shear modulus with temperature.

The G* refers to the energy of asphalt binders to resist deformation. The larger the G* value, the stronger the ability of the asphalt binders to resist deformation; that is, the greater the stiffness of the asphalt binders [30]. Figure 4 shows the changing trend of G* of fiber-reinforced asphalt binders and control asphalt binders in the temperature range from −10 °C to 65 °C. It can be seen from Figure 4 that the G* of the control asphalt binders decreases with the increase in testing temperature, and the same trend can be obtained in the curves of the other three fiber-reinforced asphalt binders.

Within the test temperature conditions from 20 °C to 65 °C, the G* of the fiber-reinforced asphalt binders is larger than that of the control asphalt binders; that is, the stiffness of the fiber-reinforced asphalt binders is improved. This increase is reasonable, where asphalt binders and fiber play a skeleton role. Among them, the increase in PPF-reinforced asphalt binders’ stiffness is the most significant. That is because PPFs have good dispersion in asphalt binders, and their surface is irregular. According to the mixing model of asphalt and fiber proposed by K.A. Liebinjieer, there is more structural asphalt in the irregular PPFs and asphalt system, which will greatly improve the stiffness of asphalt binders at high-temperature conditions [31].

However, in the temperature range between −10 °C and 20 °C, compared with the control asphalt binders, the G* of PPF-reinforced asphalt binders decreased, and the G* of LFS-reinforced asphalt binders increased significantly. It can be seen from Figure 4 that PPFs can increase the stiffness of asphalt binders under high-temperature conditions, while reducing the stiffness at low-temperature conditions.

#### 3.1.2. Phase Angle

**Figure 5:**
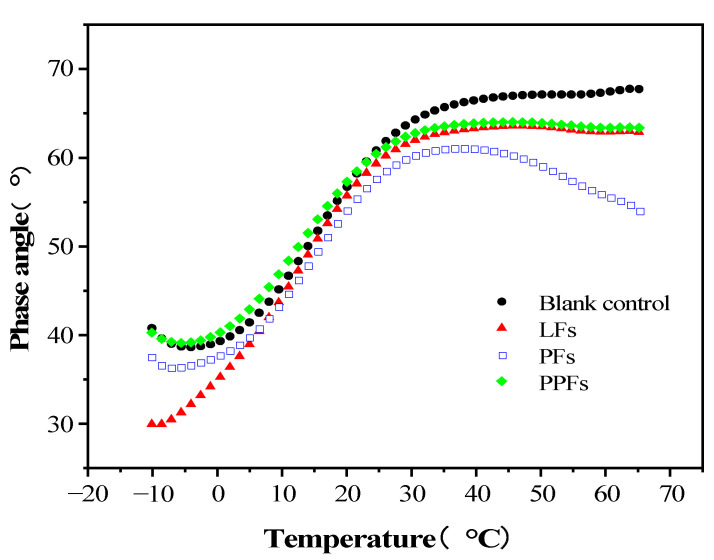
Changing trend of phase angle with temperature.

The δ indirectly reflects the size of the viscous and elastic ratio of asphalt binders itself under load. When the asphalt binders are an elastic solid, δ is 0°, and when approaching the viscous fluid, δ is 90°. Figure 5 shows the changing trend of δ of fiber-reinforced asphalt binders and control asphalt binders in the temperature range from −10 °C to 65 °C. Within the test temperature conditions from −10 °C to 65 °C, the phase angle of the control asphalt binders increase with the increase in temperature, and the same trend can be obtained in the curves of the other three fiber-reinforced asphalt binders. This is because in the process of temperature rise, the movement of polymer chains inside the asphalt binders accelerates, the viscous components inside the asphalt binders increase sharply, the elastic components decrease, and the asphalt binders change from an elastic state to a viscous flow state.

The increase in δ is the largest in the range between 5 °C and 25 °C, and the value of δ tends to be gentle after 40 °C. In the temperature range between 25 °C and 65 °C, the δ of the fiber-reinforced asphalt binders is smaller than that of the control asphalt binders; that is, the elastic properties of the fiber-reinforced asphalt binders are improved. Among them, the improvement in the elastic properties of PF-reinforced asphalt binders is the most significant. However, in the temperature range between −10 °C and 25 °C, the δ of PPF-reinforced asphalt binders increased compared with the control asphalt binders. It can be seen from Figure 5 that the addition of fiber can effectively improve the elastic properties of asphalt binders at high temperatures and delay the change process of asphalt binders to a viscous flow state. Among them, PFs have the greatest improvement in the elastic properties of asphalt binders at high temperatures.

### 3.2. Rutting Parameter and Fatigue Parameter

#### 3.2.1. Rutting Performance

**Figure 6:**
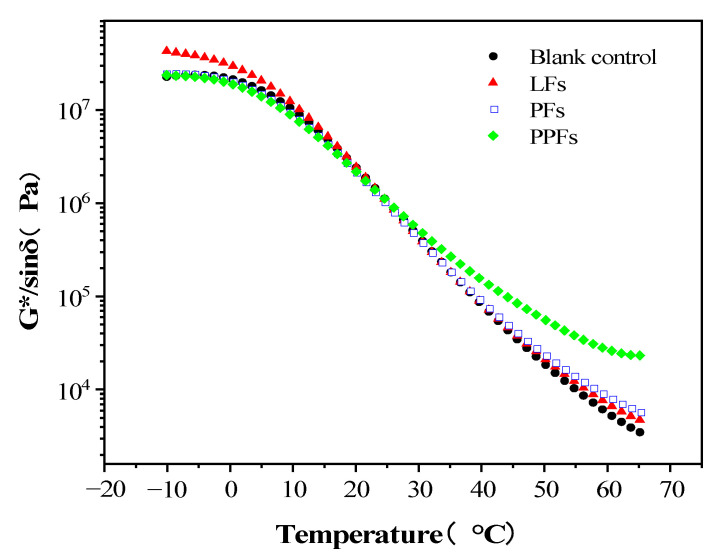
Changing trend of rutting parameter in terms of temperature.

The rutting parameter G*/sinδ can evaluate the high-temperature stability of asphalt binders. Under high-temperature conditions, the greater the G*/sinδ, the better the rutting resistance of asphalt [32]. Figure 6 shows the changing trend of the rutting parameter of fiber-reinforced asphalt binders and control asphalt binders in the temperature range from −10 °C to 65 °C. Within the test temperature conditions from −10 °C to 65 °C, the G*/sinδ of the control asphalt binders decrease with the increase in temperature, and the same trend can be obtained in the curves of PPF- and LF-reinforced asphalt binders. This is because, under high-temperature conditions, the addition of fibers will increase the stiffness of the asphalt binders and improve their elastic properties, which makes the fiber improve the rutting resistance of the asphalt binders. In the temperature range between 25 °C and 65 °C, the G*/sinδ of the fiber-reinforced asphalt binders is larger than that of the control asphalt binders; that is, the anti-rutting resistance of the fiber-reinforced asphalt binders is improved. Among them, the G*/sinδ improvement of PPF-reinforced asphalt binders is the most significant.

The existence of the fiber changes asphalt binders into structural asphalt domain materials. Structural asphalt will reduce the content of viscous components in the asphalt binders, which is why the G*/sinδ of the fiber-reinforced asphalt binders is smaller than that of the control asphalt binders at a high-temperature range. It can be seen from Figure 2 that the surfaces of PPFs and LFS are not smooth. Especially for PPFs, its surface even has a barbed structure. This allows the diffusion solvation film on the surface of PPFs and LFS to encapsulate more structural asphalt. This makes the high-temperature rutting resistance of PPF-reinforced asphalt binders better than that of other fiber-reinforced asphalt binders.

#### 3.2.2. Fatigue Performance

In order to study the effect of fiber on the fatigue resistance of asphalt binders, the fatigue parameter of G*sinδ was calculated. G*sinδ refers to the loss of the shear modulus of the binders. The larger the value, the faster the energy loss of the specimen during the loading process. The lost energy is directly related to the fatigue damage of the specimen during its loading process. The smaller the value, the slower the fatigue damage develops and the better the anti-fatigue performance [33]. G*sinδ is the test result under small strain and a small number of shear actions in the linear viscoelastic range, which can reflect the actual pavement fatigue cracking mechanical state and process to a certain extent.

**Figure 7:**
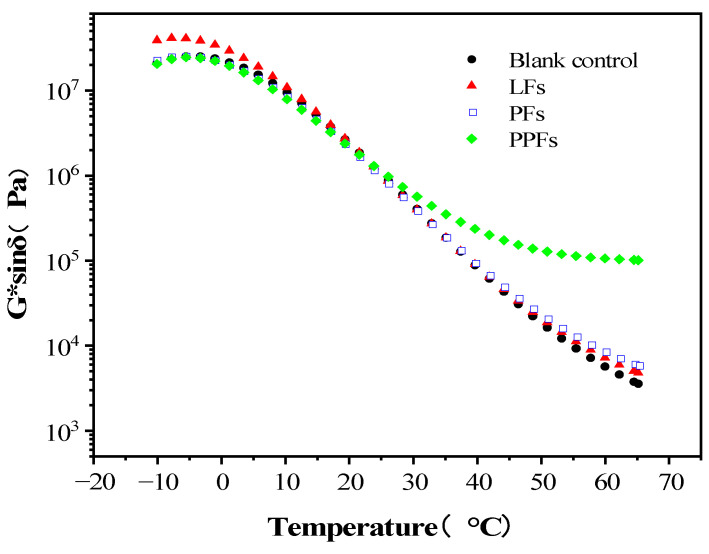
Changing trend of fatigue parameter in terms of temperature.

Figure 7 shows the change trend of G*sinδ of fiber-reinforced asphalt binders and control asphalt binders in the temperature range from −10 °C to 65 °C. Within the test temperature conditions from −10 °C to 65 °C, the G*sinδ of the control asphalt binders decreases with the increase in temperature, and the same trend can be obtained in the curves of the other three fiber-reinforced asphalt binders. In the temperature range between 25 °C and 65 °C, the G*sinδ of fiber-reinforced asphalt binders is larger than that of the control asphalt binders; that is, the addition of fiber will reduce the fatigue resistance of asphalt. Among them, the fatigue resistance of PPF-reinforced asphalt binders is the weakest.

## 4. Viscoelastic Properties of Fiber-Reinforced Mixture

### 4.1. Indirect Tensile Resilience Modulus

According to Standard Test Methods of Bitumen and Bituminous Mixtures for Highway Engineering (JTG E20-2011), the influence of fiber on the viscoelastic properties of asphalt mixtures was studied by testing the indirect tensile modulus of an asphalt mixture with Marshall specimens.

**Figure 8:**
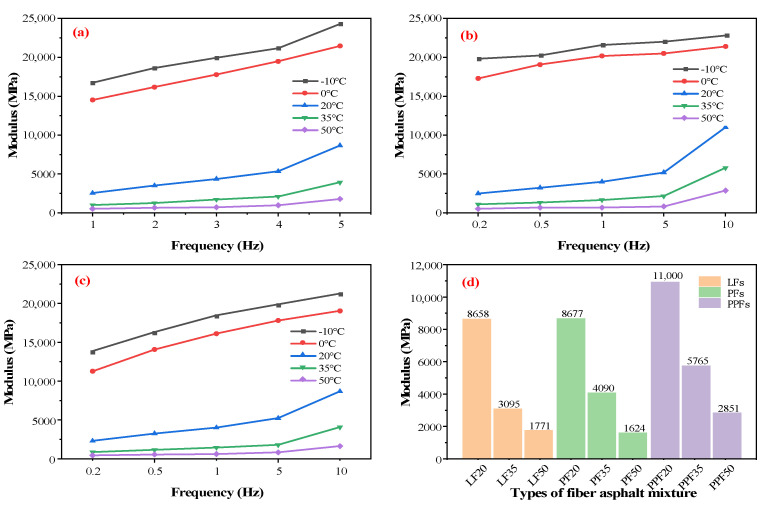
Resilience modulus of fiber asphalt mixtures: (**a**) LFs, (**b**) PFs, (**c**) PPFs. (**d**) Modulus of asphalt mixture at 10 Hz.

Figure 8 shows the indirect tensile modulus test results of the fiber-reinforced asphalt mixture. It can be seen from Figure 8a that in 0.2–10 Hz, the resilient modulus of the LF-fiber-reinforced asphalt mixture increases with the increase in frequency and decreases with the increase in temperature. The same trend is also reflected in Figure 8b,c. This is caused by the decrease in elasticity and the increase in viscosity of the asphalt mixture. At the loading frequency of 0.2–5 Hz, the resilient modulus increases slowly with the increase in frequency. At the loading frequency of 5–10 Hz, the increase in resilient modulus increases obviously.

Figure 8d shows the rebound modulus of fiber-reinforced asphalt mixtures at 20 °C, 35 °C, 50 °C, and 10 Hz. It can be seen from Figure 8d that the resilient modulus of the PPF-reinforced asphalt mixture is generally higher than that of LFs and PFs, and the resilient modulus of the PF-asphalt mixture is generally the lowest. This is mainly because the surface structure of PPFs, and its strong dispersion, increases the structural asphalt inside the PPF–asphalt mixture, so that the elastic components of the asphalt binders increase, thereby increasing the stiffness of the asphalt.

### 4.2. Master Curves of Modulus

In order to study the change degree of the rebound modulus of the fiber asphalt mixture in different frequency loading processes, a temperature of 20 °C was used as the reference temperature. The modulus frequency curve at a single temperature is translated according to the time–temperature equivalence principle, and the amount of movement needs to be calculated by the WLF equation [16]. The sigmoidal equation is defined as a general equation, which is not only applicable to the modulus and phase angle of the asphalt and asphalt mixture, but also to the compressive strength.

(1) $$\log(\alpha_{T})=\frac{C_{1}({T-T}_{\mathrm{ref}})}{C_{2}+({T-T}_{\mathrm{ref}})}$$

In the equation: α_T_ is the displacement factor at temperature T; T_ref_ is the reference temperature (°C); and C_1_ and C_2_ are constant parameters.

In this experiment, the specific parameters used to calculate the displacement factor are shown in Table 6. After moving the frequency modulus of different temperatures through the displacement factor, the data are fitted to obtain the master curve of the resilient modulus of the fiber-reinforced asphalt mixture. The model and parameters of the curve are shown in Table 7. The slope refers to the growth rate of the curve in the range of 10^−1^–10 Hz.

**Figure 9:**
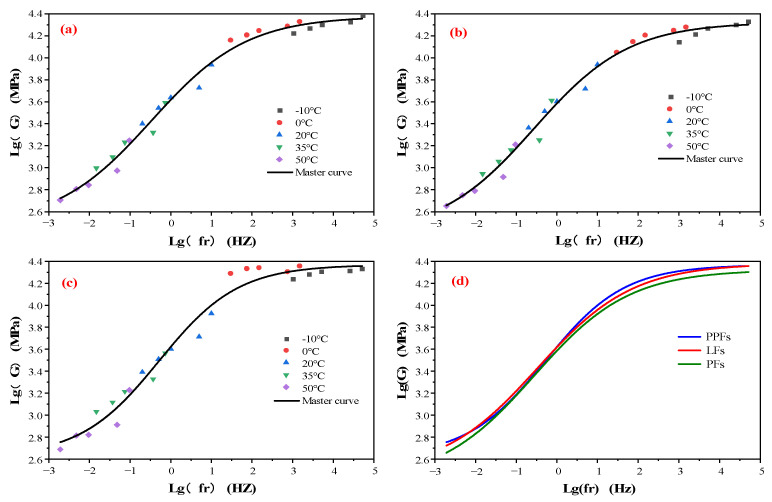
Resilience modulus of various fiber asphalt mixtures: (**a**) LFs, (**b**) PFs, (**c**) PPFs. (**d**) Master curves of fiber asphalt mixture.

Figure 9 is the master curve of the resilient modulus of the fiber-reinforced asphalt mixtures. It can be seen from Figure 9a that the resilient modulus of the LF-reinforced asphalt mixture increases with the increase in frequency, and at both ends of the curve, the increase rate tends to be gentle. The same trend can also be obtained in Figure 9b,c. It shows that the resilient modulus of the asphalt mixture has maximum and minimum values. It can be seen from Figure 9d that the master curves of the three fiber-reinforced asphalt mixtures are consistent and the linearity is very close, especially in the frequency range of 10^−1^–10 Hz. Therefore, through the linear fitting of the 10^−1^–10 Hz curve, the growth rate of the master curve of the three fiber-reinforced asphalt mixtures in this range is obtained.

It can be seen from Table 6 that the master curve slope of the PPF-reinforced asphalt mixture is the largest in the range of 10^−1^–10 Hz, indicating that it is greatly affected by frequency. In addition, both ends of the master curve of the PPF-reinforced asphalt mixture are higher, indicating that the stiffness of the PPF-reinforced asphalt mixture is large, resulting in strong high-temperature deformation resistance. The master curve of the PF-reinforced asphalt mixture is slightly lower than the other two, indicating that the stiffness of the PF-reinforced asphalt mixture is slightly lower, resulting in poor high-temperature deformation resistance. However, the low-temperature crack resistance of the PF-reinforced asphalt mixture is better. In contrast, the master curve of the resilient modulus of the LF-reinforced asphalt mixture is relatively smooth, indicating that the LF-reinforced asphalt mixture is less affected by the frequency change.

## 5. Conclusions

In this study, LFs, PFs, and PPFs were used to reinforce asphalt binders and their mixture for high-speed vehicle test field applications. The viscoelastic properties of fiber-reinforced asphalt binders and their asphalt mixture were characterized by rheological behavior and an indirect tensile resilient modulus. The following conclusions are drawn.

1. Fiber can improve the high-temperature stability of asphalt binders. The study found that the rutting parameters of the fiber-reinforced asphalt binders were greater than those of the blank control group. Among them, at 25–65 °C, the rutting parameters of PPF-reinforced asphalt binders are much larger than those of LF- and PF-reinforced asphalt binders, and the blank control group.
2. The fiber will reduce the anti-fatigue performance of asphalt binders to varying degrees while improving the anti-rutting performance of the asphalt binders. It was found that the fatigue parameters of PPF-reinforced asphalt binders were much larger than those of the other three groups at 25–65 °C. At 15–25 °C, this trend is not obvious.
3. The viscoelastic properties of fiber-reinforced asphalt binders have a great relationship with the morphology of fiber. The morphology of PPFs is short columnar, and its surface has an irregular barbed structure. This allows PPFs to be uniformly dispersed in the asphalt binders, and they have the most structural asphalt on their surface. This is the main reason for the best anti-rutting performance of PPF-reinforced asphalt binders. The flocculent morphology of LFs makes it difficult to uniformly disperse in the asphalt binders, so that the viscoelastic properties of LF-reinforced asphalt binders are not prominent.
4. From the master curve model of the resilient modulus of the fiber-reinforced asphalt mixture, it can be seen that the change trend of viscoelastic properties of fiber-reinforced asphalt binders and their mixture are basically the same. It is found that the main curves of the PPF-reinforced asphalt mixture are higher at both ends, indicating that the PPF-reinforced asphalt mixture has high stiffness and strong high-temperature stability.
5. The study found that the best choice of fiber stabilizer in a fiber asphalt mixture is a fiber with regular columnar morphology on a macro and irregular structure, or a barbed structure on a micro surface.

The new perspectives of the investigation on the topic studied are as follows:

1. Select the best form of fiber to explore the best content of fiber in a fiber-reinforced asphalt mixture.
2. The microscopic test method is used to study the combination of fiber and asphalt. The relationship between the fiber–asphalt transition zone and the performance of the fiber-reinforced asphalt mixture was discussed.

## Figures and Tables

**Table 1:** Properties of fibers.

Fiber	Length (mm)	Tensile Strength (MPa)	Density (g/cm^3^)	Melting Point (°C)	Oil Absorption (Times of Own Weight)
LFs	5	-	1.10	-	6.2
PFs	8	508	1.38	260	-
PPFs	6	450	0.91	220	-
Standard	6–12 (LFs < 6)	>270	-	>220	5–9

**Table 2:** Properties of asphalt binders.

Characteristics		Experimental Values	Standard
Penetration@25 °C, 100 g, 5 s (0.1 mm)		58.6	50–60
Ductility@5 cm/min, 5 °C (1 cm)		23.8	≥20
Softening point (°C)		79.1	≥60
Thin film oven test	Ductility@5 cm/min, 5 °C (1 cm)	17.8	≥15
	Mass loss rate (%)	+0.016	±1.0
	Residual penetration ratio (%)	75.5	≥65
Solubility (%)		99.90	≥99
Density (g/cm^3^)		1.017	-

**Table 3:** The passing rate of sieve sizes at all levels of mineral aggregate gradation.

Types	Percentage of Mass (%) through the Following Sieves (mm)									
	16.0	13.2	9.5	4.75	2.36	1.18	0.6	0.3	0.15	0.075
Upper limit	100.0	100.0	75.0	34.0	26.0	24.0	20.0	16.0	15.0	12.0
Lower limit	100.0	90.0	50.0	20.0	15.0	14.0	12.0	10.0	9.0	8.0
Gradation A(35:35:19:11)	100.0	97.3	64.0	32.1	22.3	17.5	14.9	14.0	12.7	11.2

**Table 4:** Gradation A asphalt mixture volumetric properties.

Gradation	Bitumen Aggregate Ratio (%)	Specimen Gross Density (g/cm^3^)	Maximum Theoretical Relative Density (g/cm^3^)	Air Voids (%)	Voids in Mineral Aggregate VMA (%)	Voids Filled with Asphalt VFA (%)	Coarse Aggregate Skeleton Clearance Rate VCA_mix_ (%)
A	6.4	2.479	2.572	3.6	18.2	80.1	40.7
Standard	-	-	-	3–4	≥17.0	75–85	≤VCA_DRC_

**Table 5:** DSR test parameter requirements.

Parameter	Requirement
Working form	Temperature scan
Test temperature	−10 to 35 °C, 20–65 °C
Test frequency	10 rad/s
Control mode	Strain control (5%)
Sample size	r = 4 mm, h = 2 mm and r = 12.5 mm, h = 1 mm

**Table 6:** Parameters of the displacement factor at temperature.

Parameters	T_ref_	C_1_	C_2_
-	20 °C	8.860	101.6

**Table 7:** Master curve models and parameters of resilient modulus.

Types	Models	Lg(G)_max_ (MPa)	Lg(G)_min_ (MPa)	Slope	R^2^
LFs	$y=2.475+1.904/\left(1+\frac{0.123}{10^{x}}\right)$	4.377	2.621	0.378	0.988
PFs	$y=2.396+1.927/\left(1+\frac{0.103}{10^{x}}\right)$	4.323	2.397	0.379	0.986
PPFs	$y=2.621+1.747/\left(1+\frac{0.233}{10^{x}}\right)$	4.238	2.706	0.421	0.978

## Data Availability

No new data were created or analyzed in this study. Data sharing is not applicable to this article.
